# Genetic inference of the mating system of free-ranging domestic dogs

**DOI:** 10.1093/beheco/arab011

**Published:** 2021-04-02

**Authors:** Eugenia Natoli, Roberto Bonanni, Simona Cafazzo, Daniel S Mills, Dominique Pontier, Małgorzata Pilot

**Affiliations:** 1 Canile Sovrazonale, ASL Roma 3 (Local Health Unit Rome 3), Via della Magliana 856H, 00148 Rome, Italy; 4 School of Life Sciences, University of Lincoln, Lincoln LN6 7DL, UK; 5 Université de Lyon, Université Lyon 1, CNRS, Laboratoire de Biométrie et Biologie Evolutive UMR 5558, F-69622 Villeurbanne, France; 6 Museum and Institute of Zoology, Polish Academy of Sciences, ul. Nadwiślańska 108, 80-680 Gdańsk, Poland

**Keywords:** domestication, free-ranging dogs, mating system, polygynandry

## Abstract

Domestication has greatly changed the social and reproductive behavior of dogs relative to that of wild members of the genus *Canis,* which typically exhibit social monogamy and extended parental care. Unlike a typical gray wolf pack that consists of a single breeding pair and their offspring from multiple seasons, a group of free-ranging dogs (FRDs) can include multiple breeding individuals of both sexes. To understand the consequences of this shift in reproductive behavior, we reconstructed the genetic pedigree of an FRD population and assessed the kinship patterns in social groups, based on genome-wide single-nucleotide polymorphism genotypes. Consistent with behavioral observations, the mating system of the study population was characterized by polygynandry. Instead of the discreet family units observed in wolves, FRDs were linked by a network of kinship relationships that spread across packs. However, we also observed reproduction of the same male–female pairs in multiple seasons, retention of adult offspring in natal packs, and dispersal between neighboring packs—patterns in common with wolves. Although monogamy is the predominant mating system in wolves, polygyny and polyandry are occasionally observed in response to increased food availability. Thus, polygynandry of domestic dogs was likely influenced by the shift in ecological niche from an apex predator to a human commensal.

## Teaser

In groups of free-ranging dogs, many males and females mate and produce puppies with more than one partner. Sometimes, a female delivers a litter with more than one father. This mating system contrasts with the predominantly monogamous system of the ancestor, the grey wolf. The likely cause is the access to human waste, allowing dogs to better cope with the energetic costs of reproduction leading to a higher number of reproductive animals of both sexes.

## INTRODUCTION

Due to the prevalence of social monogamy in humans and its rarity in mammals, the evolution of this mating strategy has been extensively studied (Reichard and Boesch 2003). Many studies have focused on the mechanisms leading to the emergence of monogamy (e.g., Brotherton and Komers 2003; Gavrilets 2012; Opie et al. 2013; Lukas and Clutton-Brock 2013; Jungwirth and Johnstone 2019). The conditions thought to promote the transition toward social monogamy include social intolerance among breeding females, ability of females to interfere with one another’s mating decisions, differences in fitness among females, low female density, harsh environmental conditions, and male inability to defend access to multiple females (Lucas and Clutton-Brock 2013; Jungwirth and Johnstone 2019). Identifying the factors underlying the transition from monogamy to another mating system may provide another way to understand the evolution of social monogamy, which has been rarely explored.

In mammals, social monogamy is associated with genetic monogamy, and the incidence of extra-pair paternity is low (Clutton-Brock and Isvaran 2006). Social monogamy occurs in about 9% of contemporary mammalian species and originated almost exclusively from the ancestral state where breeding females are solitary, that is, they do not form a social group with adult males or other females (Lukas and Clutton-Brock 2013). Such solitary breeding in females is found in a further 68% of contemporary species. The remaining 23% of contemporary mammalian species live in social groups containing multiple breeding females (Lukas and Clutton-Brock 2013). These social groups are typically characterized by either polygyny (i.e., males mating with multiple females within a breeding season), polygynandry (both males and females mating with multiple partners), or promiscuity (as per the latter, but without social bonds between mating individuals). Transition from social monogamy to multi-female social groups has occurred in several species, for example, banded mongoose *Mungos mungo* and Goeldii’s monkey *Calimico goeldii* (Lukas and Clutton-Brock 2013), but it is unclear what has triggered such process.

In monogamous mammals, paternal care is much more common than in nonmonogamous mammals, although it has been recorded in just 56% of the socially monogamous mammalian species, and it appears as a consequence rather than a cause of the evolution of social monogamy (Lukas and Clutton-Brock 2013; Stockley and Hobson 2016). In cooperatively breeding monogamous carnivores, the presence of both biparental care and alloparental care provided by nonreproductive group members is associated with larger litter size, higher litter growth rate, and higher offspring survival (Creel and Creel 1991; Moehlman and Hofer 1997; Stahler et al. 2013; Ausband 2019). It has been argued that during the evolution of these species, the assistance provided by fathers and nonreproductive alloparents allowed greater energetic investment in gestation and postnatal care of offspring to the point that mothers could no longer meet the energetic requirements of reproduction without help (Creel and Creel 1991). However, specific ecological conditions may promote a transition from monogamy to another mating system. According to the “polygyny threshold model,” when resource availability increases above a given threshold, a female mated with a polygynous male will be able to raise as many offspring as a female mated with a monogamous male despite receiving reduced paternal assistance, which can lead to a shift in the mating system (Orians 1969). Moreover, a shift toward polyandry due to increased food availability and population density has also been described in several socially monogamous birds (Griffith et al. 2002) and canids (Baker et al. 2004; Carmichael et al. 2007; Iossa et al. 2008; Macdonald et al. 2019).

Anthropogenic environments are characterized by abundant food resources, leading to high population densities of animals living as human commensals. Therefore, domesticated animals provide excellent models to study transitions between monogamous and polygamous mating systems. Domestication is associated with rapid and extensive phenotypic changes, resulting from artificial selection and adaptations to human-modified habitats (Larson and Fueller 2014). Many domestic animals living as human commensals in anthropogenic environments experienced changes in their social and mating behavior (Hulme-Beaman et al. 2018). Such changes occurred in the case of domestic dogs (*Canis lupus familiaris*) that live in social groups consisting of multiple breeding males and females, unlike their monogamous wild ancestor, the gray wolf (*Canis lupus*; Lord et al. 2013; Bonanni and Cafazzo 2014; Marshall-Pescini et al. 2017). This change in the mating system occurred at a very fast rate compared with transitions associated with the speciation process, given that the onset of dog domestication has been estimated at about 25 000 – 40 000 years ago (reviewed in Freedman and Wayne 2017), preceding the Neolithic transition that resulted in increased availability of human food waste. As all wild canids display social monogamy (Macdonald et al. 2019), this change in the mating system was specific to domestic dogs and, therefore, can be attributed to the ecological circumstances associated with domestication, which are relatively well established (e.g., Freedman and Wayne 2017; Marshall-Pescini et al. 2017). The comparison between the gray wolf and the domestic dog as its direct descendant provides a rare opportunity to study a transition between mating systems where the monogamous ancestral state is known rather than inferred based on phylogeny.

About 75% of the global dog population is free-ranging and unconstrained in their mate choice (Gompper 2014). Eurasian free-ranging dog (FRD) populations are not a product of admixture between breeds but constitute a distinct and older genetic group (Pilot et al. 2015; Shannon et al. 2015). Behavioral traits of modern FRDs have not been modified by recent artificial selection imposed on pure-bred dogs, and, therefore, it can be expected that their mating system will reflect adaptive processes occurring during their evolution in anthropogenic environments.

FRDs vary greatly with respect to their degree of association with humans, living environment and diet. Some FRDs are affiliated to humans and may be used for hunting and livestock guarding, whereas others avoid social interactions with humans (Woodroffe et al. 2007; Boyko and Boyko 2014; Bonanni and Cafazzo 2014). FRDs living in urban environments subsist primarily on anthropogenic food sources, either provided intentionally or in the form of waste. FRDs living in rural areas include natural prey in their diet, but populations that are independent of anthropogenic food sources are rare (Vanak and Gompper 2009; Ritchie et al. 2014). There are few documented cases of truly feral dog populations, which are demographically and ecologically independent of humans over the long term; known examples include Australian dingoes, New Guinea highland wild dogs, and a population from Isabela Island, Galapagos (Reponen et al. 2014; Surbakti et al. 2020; Zhang et al. 2020). Even though typical FRDs are dependent on human-derived food, they can express their natural social and reproductive behavior without the constraints imposed by humans. Thus, they are subject to natural and sexual selection pressures similar to those affecting wild canids (Pilot et al. 2016).

FRDs display flexible social organization and can form social groups (packs) of varying sizes and stability of membership (Bonanni and Cafazzo 2014). While a typical pack of gray wolves (*Canis lupus*) consists of a single monogamous breeding pair and their offspring from multiple breeding seasons (Mech and Boitani 2003), FRD packs can include multiple breeding individuals of both sexes (Daniels 1983; Boitani and Ciucci 1995; Boitani et al. 2016; Pal 2011; Bonanni and Cafazzo 2014). Behavioral studies on FRDs frequently report a promiscuous mating system, but monogamous pairs have also been observed (Daniels 1983; Gipson 1983; Pal 2005, 2011; Cafazzo et al. 2014), and males seem to vary considerably in their degree of parental investment (Pal 2005; Paul and Bhadra 2018). Studies on Indian FRDs showed considerable variation in male copulation success (Pal et al. 1999), and a study of a large FRD pack in Italy showed that both male copulation success and female reproductive success varied considerably among breeding individuals and were significantly affected by interrelated variables, such as dominance rank, age, leadership, and earlier affiliative interactions between males and females (Cafazzo et al. 2014).

While the social and reproductive behavior of FRDs has been well described in behavioral studies, the genetic mating system of domestic dogs remains unknown. In this study, we fill this knowledge gap by reconstructing the genetic pedigree of an FRD population and comparing it with affiliations of individuals to packs. We hypothesize that the energetic constraints on reproduction, which make female wolves (as well as other wild canids) dependent on paternal and alloparental care (Creel and Creel 1991; Moehlman and Hofer 1997; Macdonald et al. 2019), were relaxed in FRDs as a result of access to anthropogenic food, which is relatively abundant all year round and can be obtained with reduced energy expenditure relative to natural prey. Easier access to food could also reduce social intolerance among breeding females. Moreover, high population density resulting from access to anthopogenic food increased the chances of finding multiple mates for both males and females. We predict that the new conditions resulting from the exploitation of anthropogenic food sources initiated a transition from the social monogamy typical of all wild canids toward a mating system characterized by male and female polygamy.

## METHODS

### Study population

**Figure 1:**
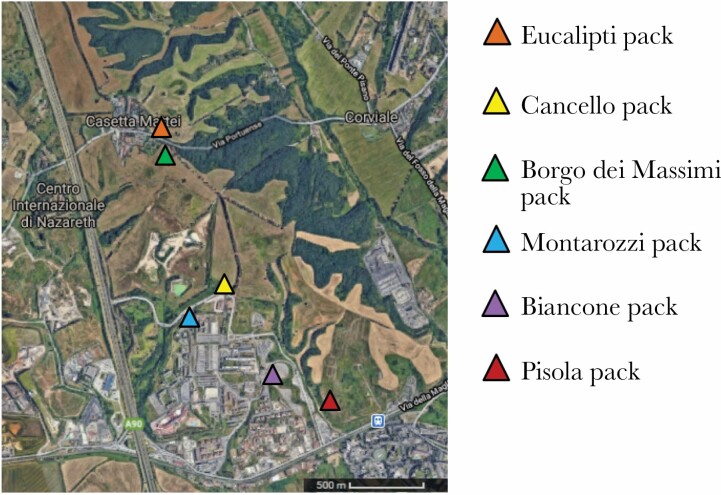
Distribution of the feeding sites used predominately by each individual dog pack studied.

The study was carried out in a suburban area in the southwestern outskirts of Rome, Italy, which covered about 300 ha and was delimited to the north, west, and south by roads with heavy traffic and to the east by cultivated areas. The area was split by another road into two sectors, one in the southwest and another in the northeast. The southwest sector was urbanized, although not densely populated. The northeast sector was mainly occupied by a nature reserve (“Tenuta dei Massimi”). The habitat in the reserve consisted mainly of open grassland with interspersed wooded areas. FRDs were free to move across every sector of the study area. They used the wooded areas of the reserve to find resting sites, refuges, and dens for puppies. However, they spent considerable time around both the central road crossing the study area and another road crossing the nature reserve in the northern sector to feed on the abundant food brought by volunteer dog caretakers every morning. Food (mainly meat from a slaughterhouse) was placed, together with water, at specific feeding sites located close to the two roads (Figure 1). The food was supplied in such quantity that some uneaten food remained every day. The practice of intentionally feeding FRDs is widespread in many parts of the world, although the amount of food provided varies and not all FRDs are intentionally fed (e.g., Gipson 1983; Chang 2012; FAO 2014; Serpell 2016; Capellà Miternique and Gaunet 2020).

The dog population was monitored regularly by our working group from April 2005 to April 2011. Detailed population censuses were carried out periodically in 2005–2006, 2007–2008, and 2010–2011, by enumerating all individually recognized dogs that approached the road to feed. Intensive behavioral studies were carried out on a 27-member pack from April 2005 to May 2006 and on other three packs (ranging in size from 3 to 15 individuals) from May 2007 to September 2008 (for details, see Bonanni et al. 2010; Cafazzo et al. 2010). Individuals were considered to belong to the same pack if they traveled, rested, and defended resources as a cohesive unit (Cafazzo 2007; Bonanni and Cafazzo 2014), thus fitting the definition of a canid pack (Mech 1970).

Population size was relatively stable across years and, in May 2011, when the sample collection for this study began, the FRD population comprised 97 animals (53 adult males, 1 subadult male, 38 adult females, and 5 individuals whose sex was not determined). The sex ratio in the population was male-biased, although the bias was small relative to that reported in some other populations (e.g., Ortolani et al. 2009; Mustiana et al. 2015). Out of 92 individuals whose sex was ascertained, 23 individuals (25%) had been neutered before the study began; this included 10 out of 54 males (18.5%) and 13 out of 38 females (34.2%). Additional individuals were neutered at the time of the sample collection (see below), but this did not affect the mating patterns inferred from the genetic data, as only patterns prior to the sample collection (and thus neutering) could be inferred.

The adult dogs were medium to large sized: 27 adult dogs captured weighted 34.10 kg on average (males 33.58 kg; females 34.82 kg) and had an average height at the withers of 66.50 cm (males 67.00 cm; females 65.82). All dogs in the population were individually recognized through the identification based on their coat color and pattern, fur length, body size, and sex. The phenotypic variation in this population (see Supplementary Figure S1) was not large but sufficient to distinguish individuals.

**Figure 2:**
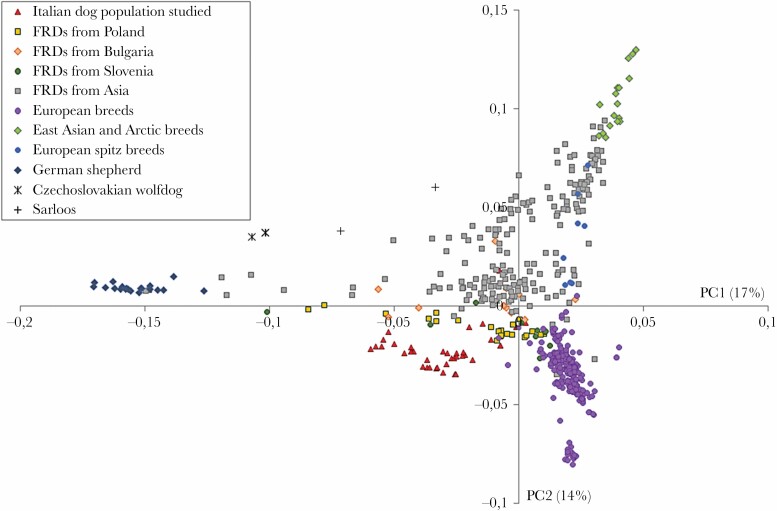
Genetic differentiation between the Italian FRDs studied (red triangles) and other FRD populations, as well as pure-bred dogs, reconstructed using the principal component analysis. Data on other populations and breeds were obtained from Vaysse et al. (2011) and Pilot et al. (2015).

With very few exceptions, dogs were not socialized to humans, that is, they displayed strong and persistent avoidance responses to humans, despite the human food provisioning. The comparison of the genome-wide single nucleotide polymorphism (SNP) genotypes of dogs from this population to FRDs from across Eurasia and pure-bred dogs (Vaysse et al. 2011; Pilot et al. 2015) showed that the population studied does not constitute a mixture of breeds and shows genetic similarity to other European FRD populations (Figure 2; for details, see Supplementary Results).

An estimate of the density was about 30 animals/km^2^, which is in-between that of dog populations living in strictly urban areas, where the density is higher, and that of dogs living in more natural environments, where the density is much lower (see Boitani and Ciucci 1995 for review). The population was not isolated by any geographic barriers and immigration/emigration from/to the study area was not prevented in any way.

Almost all dogs inhabiting the study area lived in packs with stable membership (Bonanni and Cafazzo 2014; Bonanni et al. 2017). Each pack comprised core members who stayed together for years plus transient members who were loosely associated and might join or leave on a monthly basis. Most packs fed mainly, but not exclusively, at a specific feeding site (Figure 1), although some packs habitually used multiple feeding sites. Since the study area was relatively small, most feeding sites were located within a few hundred meters of each other.

We obtained tissue samples from individuals who were immobilized within the framework of a neutering program implemented by the Municipality of Rome and by the Veterinary Public Service Rome 3 of Rome in compliance to the Italian National Law no. 281/1991. The samples, which were collected between May 2011 and November 2013, included ovaries or testes from 26 adults and 2 juveniles who were successfully captured, and 18 fetuses from three early pregnancies. These 46 individuals belonged to seven packs (excluding 4 individuals with unknown pack affiliation; Table 1), although the majority of them (89%) belonged to three adjacent packs. Sample collection complied with Italian regulations on the ethical treatment of stray dogs. The study obtained ethical approval from the University of Lincoln (CoSREC365).

**Table 1:** Information about individuals studied. FS, full sibling; MHS, maternal half-sibling; PHS, paternal half-sibling

Dog identity	Pack and no. of pack members	Gender	Date of capture	Age at capture	Known kin
Fifa	Cancello 24	F	23.12.2013	Between 4 and 5 years, older than her siblings Spider and Bella, younger than Schiva and Virginia	FS of 4 ind. of this pack and of Laura (Eucalipti pack)
Spider	Cancello 24	M	04.01.2012	Less than 5 years, younger than his siblings Fifa, Schiva, Virginia, and Bella	FS of 4 ind. of this pack and of Laura (Eucalipti pack)
Bella	Cancello 24	F	23.02.2013	Between 3 and 4 years, older than her siblings Spider, younger than Fifa, Schiva, and Virginia	FS of 4 ind. of this pack and of Laura (Eucalipti pack)
Petto	Cancello 24	M	03.07.2012	Around 3 years, same age as his sister Femmina near	Son of Bella
Emma	Cancello 24	F	26.10.2012	Around 5 years	Mother of 2 fetuses sired by ♂2; MHS of ID36 and ID40
Virginia	Cancello 24	F	04.04.2012	More than 5 years, older than her siblings Fifa, Schiva, Spider, Laura, and Bella	FS of 4 ind. of this pack and of Laura (Eucalipti pack)
Schiva	Cancello 24	F	04.04.2012	More than 5 years, older than her siblings Fifa, Spider, Laura, and Bella, younger than Virginia	FS of 4 ind. of this pack and of Laura (Eucalipti pack)
Angelo	Cancello 24	M	09.12.2011	Between 4 and 5 years, same age as his sister Sofia	FS of Sofia (Borgo dei Massimi pack)
ID49	Cancello 24	M	26.11.2013	Young adult	Son of Fifa
ID36	Cancello 24	M	26.11.2013	4 months old	MHS of Emma
ID40	Cancello 24	F	19.11.2013	4 months old	MHS of Emma
Biancone	Biancone 4	M	25.05.2011	More than 5 years	Son of Schiva
Macchiato	Montarozzi 5	M	17.05.2011	More than 5 years	PHS of Nello
Mirko	Pisola 3	M	13.12.2011	Between 2 and 3 years	PHS of ID20 (fetus); MHS of Bo
Laura	Eucalipti 15	F	26.01.2012	Between 2 and 3 years, younger than her siblings Fifa, Schiva, and Virginia	FS of 5 individuals of Cancello pack
Marco	Eucalipti 15	M	23.02.2012	Around 2 years, same age as his brother Fred	FS of Fred (Borgo dei Massimi pack) + 6 fetuses, HS of ID19 and ID20 (fetuses)
Bo	Eucalipti 15	M	09.12.2011	Around 4 years	MHS of Mirko, father of Claudia and 7 fetuses of Sofia
Bernardo	Eucalipti 15	M	07.03.2012	More than 5 years	MHS of Sofia (Borgo dei Massimi pack) and Angelo (Cancello pack); PHS of Antonio
Claudia	Borgo dei Massimi 11	F	14.03.2012	Fully adult	Daughter of Bo (Eucalipti pack)
Antonio	Borgo dei Massimi 11	M	04.01.2012	Between 2 and 3 years	MHS of Claudia and PHS of Sofia and Angelo (Cancello pack)
Snella	Borgo dei Massimi 11	F	14.03.2012	Less than 5 years	Mother of 6 fetuses and of Marco and Fred, all sired by ♂3, one fetus sired by ♂4 and one fetus sired by ♂5
Fred	Borgo dei Massimi 11	M	09.12.2011	Around 2 years, same age as his brother Marco	FS of Marco (Borgo dei Massimi pack)
Sofia	Borgo dei Massimi 11	F	12.01.2012	Between 4 and 5 years, same age as her brother Angelo	FS of Angelo (Cancello pack); MHS of Bernardo (Eucalipti pack)
Duca	Borgo dei Massimi 11	M	24.02.2013	Between 2 and 3 years	Son of Nello
Maremmano Lallo	Unknown	M	12.04.2012	More than 5 years	No close relatives
Maremmano Nello	Unknown	M	12.04.2012	Around 4 years	Father of Duca
Femmina nera	Unknown	F	19.07.2012	Around 3 years, same age as her brother Petto	Daughter of Bella (Cancello pack)
ID29	Unknown	F	19.11.2013	Unknown	No close relatives

### Reconstruction of genetic kinship patterns within the study population

DNA extraction from the tissue samples was carried out using DNeasy Blood & Tissue Kits (Qiagen). The samples were genotyped at 360K SNP loci using Axiom Canine HD Genotyping Array (Thermo Scientific). We used Plink1.9 software (Chang et al. 2015) for the filtering of the SNP loci (see below). We removed from the analysis two individuals with more than 10% missing data. The final data set included 44 individuals: 10 adult females, 15 adult males, 2 juveniles (4 months old), and 17 fetuses from three litters.

Kinship relationships among individuals were estimated using complementary methods based on estimates of pair-wise identity by descent (IBD) coefficient (Primus software; Staples et al. 2014) and based on patterns of allele sharing across individual loci (Colony, Wang 2013; Cervus, Kalinowski et al. 2007). Due to the high complexity of the kinship relationship in the study population, we used the combined results from these three programs to obtain the reliable pedigree (family tree) reconstruction.

Primus software was used to estimate the kinship relationships in the study population based on the pair-wise IBD estimates obtained in Plink. IBD estimates were calculated for 140 061 loci distributed across all 38 autosomal chromosomes. This set of loci was obtained after filtering the data set for loci that were invariable for the analyzed population or showed very low variability (Minor Allele Frequency [MAF] <0.01), as well as loci having more than 20% missing data for the study population.

**Figure 3:**
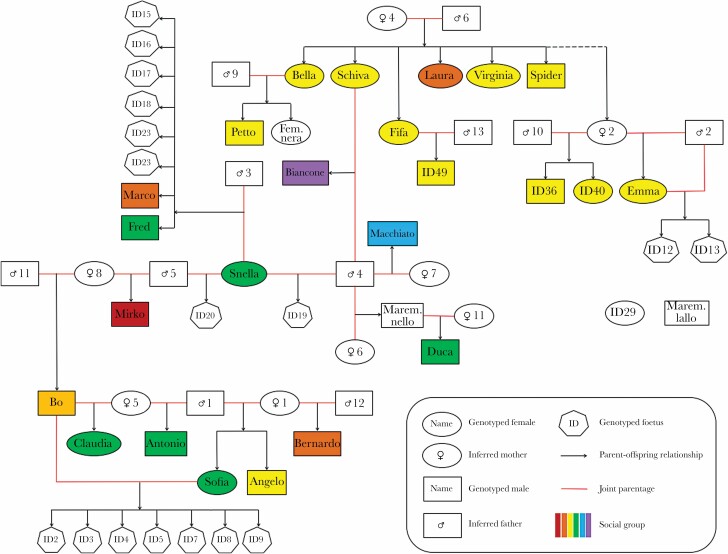
Pedigree of the study population. Individuals labeled with ♀and ♂ symbols are unsampled individuals inferred as parents based on the full-sibling or half-sibling relationships of their offspring. Numbers next to these symbols are used to distinguish different unsampled individuals; their order does not have any biological meaning. Individuals labeled with “ID” symbol are sampled individuals, who were either fetuses or pups/subadults who were not yet named. The kinship relationships were reconstructed in Colony, except the full-sibling relationship of the female ♀2 to six sampled individuals, which was inferred in Primus based on the kinship data available for that female’s offspring. Due to the high complexity of the pedigree, the vertical axis of the graph does not represent time. Two individuals that are not linked to other individuals in the graph do not have any first- or second-degree relatives in the population. Membership in packs is marked with color. The lack of color means that the pack membership of an individual is unknown.

Based on the IBD estimates, Primus identified pairs of first-, second-, and third-degree relatives (which include first cousins as well as great-grandparental and great-avuncular relationships). We ran the software with default options, with one exception. We assumed that the maximum number of generations between two individuals that produce offspring is 3. Primus typically reconstructs most likely pedigrees based on the inferred pair-wise kinship relationships. However, in this case, the software failed to resolve the pedigrees due to their high complexity. Many individuals were inferred to have offspring with multiple mating partners (e.g., a female named Snella had offspring with three different males), which made it difficult to clearly present the kinship relationships and, at the same time, distinguish individuals belonging to different age cohorts by using the vertical axis to reflect time. This resulted in the complexity of the pedigree (see Figure 3 for the pedigree reconstruction based on the combined inference from all the software used).

Cervus software was used to assign parentage based on allele-sharing patterns. The same information was obtained from Colony, but because the two programs use different algorithms of parentage assignment, the Cervus results were used to validate the parentage assignment from Colony. The Cervus analyses of maternity and paternity were based on a set of 2760 loci, which was obtained by pruning the data set used in Primus from loci in linkage disequilibrium (*r*^2^ < 0.1) and retaining only the loci with MAF higher than 0.45. The analysis of parent pairs was based on 1440 loci, because the software could not complete it for the larger number of loci due to the computational complexity. This data set was obtained from the previous one by retaining only the loci with no more than 2% of missing data. The simulations of parent–offspring pairs and trios of two parents and an offspring, based on the allele frequencies in the study populations, were used to determine the confidence levels for the assigned parentage. We simulated 10 000 offspring, 15 fathers and 9 mothers (corresponding to the number of adult individuals sampled). Two confidence levels were applied, strict (95%) and relaxed (80%), but all the parent–offspring pairs and trios identified here were assigned a strict confidence level. Cervus frequently identifies both directions of the parent–offspring relationship to be equally valid (e.g., a pair of individuals can be identified as both a mother–son pair and a father–daughter pair). Therefore, the age data were used whenever available to exclude parentage assignments inconsistent with age. We were able to exclude all conflicting parentage assignments (i.e., situations when both directions of parent–offspring relationships were inferred) based on the combination of age data and the inference from Colony (see below).

Colony software was used to assign parentage to individuals and identify groups of full and half-siblings. Colony uses the maximum likelihood approach to identify parentage based on allele-sharing patterns rather than using the estimates of relatedness averaged across loci and, therefore, may be more accurate in distinguishing between relationships of the same degree (e.g., parent–offspring vs. siblings). Moreover, Colony uses the sibship inference to establish the direction of parent–offspring relationships (e.g., if A–P and B–P are parent–offspring pairs with an unknown direction of the relationship and A and B are full sisters, this implies that P must be their parent, since both of them cannot be P’s mothers). Colony can also identify groups of full and half-siblings even in the absence of one or both parents in the data set. The Colony analysis was based on the same set of 1440 loci that was used in Cervus for the parent-pair assignment. The analysis was run in three replicates, applying the full-likelihood method. We assumed the possibility of polygamy for females and males (which does not prevent the detection of monogamous pairs if present) and the possibility of inbreeding. Information about the known mothers of the fetuses was provided as an input. All sampled individuals were included in the offspring list.

The results obtained from the above analyses were compared for consistency to create the reliable pedigree. Next, we assessed the distribution of individuals representing genetic families within and among packs in order to reconstruct the patterns of group composition and dispersal.

## RESULTS

The PI_HAT coefficient, showing the average proportion of alleles identical by descent across the loci for two individuals compared, ranged from 0 to 0.808 (excluding a pair of monozygotic twins, where it was equal to 0.999), with an average of 0.316 (standard deviation [SD] = 0.161). The within-individual inbreeding coefficient was negative for 34 (77%) individuals and positive for the remaining 10 individuals; its average value was −0.140. The population-level estimates of the inbreeding coefficient (F_IS_) were −0.184 based on heterozygosity estimates in Plink and −0.177 based on the Colony estimate. The effective population size, estimated assuming nonrandom mating, was 32 (95% confidence interval = 20–55).

The pedigree reconstructed based on the Colony results provided the most complete information, including the inference of parentage, full-sibling and half-sibling relationships, and the inference of unsampled parents (see Figure 3). Cervus provided only the inference of parentage. Primus provided inference of kinship relationships between pairs of individuals up to the third degree but could not correctly distinguish between parent–offspring and full-sibling relationships. However, Primus—unlike Colony and Cervus—directly infers more distant kinship relationships, for example, grandparent–grandchild and aunt/uncle-niece/nephew; therefore, it could infer a full-sibling relationship of an unsampled mother of three sampled individuals (♀2 in Figure 3) to six other sampled individuals based on the kinship data available for that female’s offspring. This is the only part of the reconstructed pedigree that is not based on Colony results. We compared the Cervus and Primus results against the Colony results and found them highly consistent, the only discrepancies being associated with distinguishing between parent–offspring pairs and full siblings (Supplementary Table S1). Therefore, the pedigree based on the Colony results is also supported by the inference from the two other software.

The reconstructed pedigree (Figure 3) shows a pattern consistent with polygynandry. We identified 27 individuals who had at least one offspring among genotyped individuals (14 females and 13 males). Seven of them were genotyped and 20 were inferred based on the full-sibling and half-sibling relationships between their offspring. These 27 individuals formed only 20 parent pairs because some individuals were part of multiple parent pairs. For five females and four males, we found evidence of mating with more than one individual. The lack of such data for other individuals does not imply their monogamy and does not inform us about their mating patterns, given that our sampling did not cover the entire population. The maximum number of mating partners identified was three for females and four for males. Maternal and paternal half-siblings were common in the population, which is consistent with polygynandrous mating. The inferred cases of polyandrous mating included a litter of one female (Snella) fathered by three different males. In other cases of polyandrous and polygynous mating, we cannot conclude whether mating with different individuals occurred within a short period or across longer time. However, the adult mortality in this population was low (about 10–15% per year) and adult dispersal was mostly between packs within the population (Cafazzo S, Bonnani R, unpublished data). Therefore, it is unlikely that mating with a new individual took place only after the earlier mating partner died or disappeared.

Most of the sampled individuals formed a large group joined by a network of first- and second-degree relatedness, except two individuals unrelated to the others (named “ID 29” and “Maremmano Lallo”; Figure 3). “Maremmano Lallo” was presumably abandoned by humans in the study area, which explains the lack of kinship relationships with other individuals. The maximum independent set identified in Colony, which denotes a set of individuals who are not related (up to the third-degree relatedness), consisted of only three individuals.

Of the three litters that were sampled as fetuses, one litter consisted of seven full siblings fathered by a male having a lower level of relatedness to the mother than average in the population (PI_HAT = 0.241). The second litter of two full siblings was fathered by their grandfather; this was the only detected case of incest. These two fetuses had the highest internal inbreeding coefficient of all individuals from the population (0.162 and 0.262, respectively). The third litter consisted of half-siblings fathered by three males. Six fetuses had a common father, while the remaining two fetuses were fathered by a different male each. These last two fetuses had relatively high internal inbreeding coefficient (0.085 and 0.092, respectively), implying that their fathers were related to their mother. The male who fathered six fetuses previously produced another litter with the same female. This parent pair was unrelated, as all their offspring had negative internal inbreeding coefficients. Another, inferred parent pair produced offspring together in at least two different years. This parent pair was also unrelated.

The individuals studied belonged to several packs (Figure 3; Table 1). One pack (“Cancello pack,” marked in yellow in Figure 3) included five sampled full siblings of different age with the sixth sibling inferred from the pedigree analysis, as well as two adult offspring and three juvenile offspring of three of the female siblings. Another pack (“Borgo dei Massimi,” marked in green) included a male with a maternal half-sister and a paternal half-sister, as well as a mother with an adult son. Each of the packs where multiple individuals were genotyped also included unrelated individuals. The average PI_HAT coefficients in these three packs were 0.317 (SD = 0.186), 0.354 (SD = 0.122), and 0.448 (SD = 0.126), respectively, and did not differ significantly from the population average. We also found links of close relatedness between individuals from different packs, indicating short-distance dispersal. This included two cases of full siblings and one case of maternal half-siblings living in different packs. One pack included two confirmed breeding females (i.e., with their offspring identified) and another pack included three breeding females.

## DISCUSSION

Our results are consistent with the prediction of polygamy in both male and female dogs, that is, they provide genetic evidence of polygynandry in the study population. We demonstrated the presence of multiple breeding individuals of both sexes within packs. Moreover, one-third of dogs (both males and females) identified as parents of genotyped individuals produced offspring with more than one partner. The lack of such data for other individuals does not imply their monogamy, given that our sampling did not cover the entire population. Thus, we can neither confirm nor exclude the possibility that some monogamous pairs may be present in the population. Furthermore, to our knowledge, we have detected the first genetically documented case of multiple paternity within a litter of FRDs. These results are consistent with behavioral data collected in this (Cafazzo et al. 2014) and other populations of FRDs (Daniels 1983; Gosh et al. 1984; Pal et al. 1999; Pal 2005, 2011), suggesting that their mating system is characterized by polygynandry. However, it cannot be ruled out that some females mated with only one partner, given that all puppies from two litters were sired by a single male. Behavioral observations of a large dog pack that was living in our study area (not included in this genetic study) showed that most pack members mated with more than one partner during 1 year of observation, but some individuals of both sexes were observed mating with just one partner (Cafazzo et al. 2014).

Behavioral studies showed that both female and male FRDs exhibit mate choice, and both male copulatory success and female reproductive success showed considerable asymmetries within packs (Gosh et al. 1984; Pal et al. 1999; Pal 2011; Cafazzo et al. 2014) and increased with dominance rank: high-ranking males copulated more frequently than low-ranking males, and offspring of high-ranking females had higher survival rates to reproductive age compared to offspring of low-ranking females (Cafazzo et al. 2014). However, male copulatory success does not necessarily correspond with reproductive success because the latter can also be affected by sperm competition when females mate with multiple males during a single estrus period (Hulme-Beaman et al. 2018). In our study, one male dog sired six out of eight puppies of a litter, with the other puppies sired by two other males, an outcome that could reflect sperm competition. In two other litters, we detected a single father, a pattern that may be a consequence of mating monopolization at the time of conception by a high-ranking male dog or sperm competition with a single winner. One of the two male dogs who monopolized paternity in these litters (i.e., “Bo”; Figure 3) was the second largest dog ever measured in the study area (47 kg compared to the average of 34 kg; Natoli et al. unpublished data), and it is likely that body size can affect the acquisition of dominance rank in FRDs (Bonanni et al. 2017). A dog who monopolized paternity in the second litter was the father of the litter’s mother. However, incest must have been rare in the study population, given that the population-average inbreeding coefficient as well as within-individual inbreeding coefficients of most individuals were negative. In wolves, incest is also rare and occurs when the pack structure is unstable due to intense hunting or when dispersal opportunities are limited (Smith et al. 1997; Vila et al. 2003; Jędrzejewski et al. 2005; vonHoldt et al. 2008).

In a large pack of FRDs from our study population, male–female affiliative bonds were identified as one of the main variables fostering copulation (Cafazzo et al. 2014) and long-lasting social bonds among pack members were common in this population (Bonanni and Cafazzo 2014; Bonanni et al. 2017). Here, we detected two male–female pairs that produced offspring in at least two different years, which could reflect a long-term affiliative bond between mates. Nevertheless, this does not imply a monogamous mating strategy because one of these pairs produced a litter in which paternity was shared between three males (female—Snella; males—♂3, ♂4, ♂5; Figure 3). Long-term male–female affiliative relationships are known to promote copulation also in other species that exhibit a polygynandrous mating system (e.g., spotted hyenas, *Crocuta crocuta,*East et al. 2003; chimpanzee, *Pan troglodytes,*Gomes and Boesch 2009).

The polygynandrous mating system inferred for the study population is consistent with behavioral observations in FRDs but contrasts with the predominantly monogamous mating system of gray wolves (Table 2) and other wild canids. However, mating and social systems in both wolves and dogs are flexible. Although the core of the wolf pack always consists of a breeding pair and their offspring, some packs include unrelated, subordinate males (Lehman et al. 1992; Jędrzejewski et al. 2005). Multiple breeding females are occasionally reported in wolf packs (Mech and Boitani 2003; Ausband 2018, 2019; Sidorovich and Rotenko 2019), and their frequency increases with population density and pack size (Ausband 2018; Sidorovich and Rotenko 2019), which, in turn, are regulated by food availability and foraging strategy (Macdonald et al. 2019). Polyandry in the form of “sneaker” males has also been recorded in wolf packs from Idaho and Yellowstone, where such males fathered about 13% of pups (Ausband 2018). In turn, a single female breeder has been reported for an FRD pack in Alaska living under extreme environmental pressures (Gipson 1983). Unlike the FRDs of our population, these Alaskan dogs were not intentionally fed by humans and, although they had access to a garbage dump, they frequently preyed on wildlife (Gipson 1983). By comparison, Australian dingoes, who descended from domestic dogs and have readapted to natural environments (Zhang et al. 2020), display a flexible mating system with both long-term monogamy and polygynandry observed in different individuals (Tatler et al. 2020).

**Table 2:** Comparison of the mating systems of gray wolves and domestic dogs. Data on wolves based on Lehman et al. 1992; Jędrzejewski et al. 2005; vonHoldt et al. 2008; Caniglia et al. 2014; Ausband 2018, 2019; Sidorovich and Rotenko 2019. Data on dogs based on this study

Trait	Grey wolf	Domestic dog
Litter paternity	Single	1–3 fathers
Multiple litters produced by the same parent pair	Frequently	Observed but probably less frequent than in wolves
Group affiliation of parents	The same group, with the exception of “sneaker” males breeding with females from different groups	Can remain in different groups before and after pups’ birth
Multiple breeding females within groups	Rare in stable populations but may be common in growing or heavily hunted populations	Frequent
Retention of adult offspring in natal groups	Frequent	Frequent
Presence of unrelated individuals within groups	Less frequent but not uncommon^a^	Frequent
Maternal and paternal half- sibling relationships	Rare	Frequent
Dispersal among groups within the same area	Frequent	Frequent

^a^This excludes individuals forming the dominant breeding pairs, which are typically unrelated.

Social monogamy is also typical for all other wild Canidae, but deviations from this predominant pattern are known for many species (e.g., red fox, Baker et al. 2004; Arctic fox, Carmichael et al. 2007; bat–eared fox, Wright et al. 2010; African wild dog, Spiering et al. 2010). Mating strategies of canids vary depending on the abundance and distribution of food resources, supporting the importance of feeding ecology in shaping mating systems (Noren et al. 2012; Tallents et al. 2012). For example, in the red fox, transitions from social monogamy to polygamy can occur in ecological conditions characterized by abundant food and high population density, which include urban environments (Baker et al. 2004; Iossa et al. 2008).

Taken together, these findings show that wolves and other wild canids as well as FRDs maintain behavioral plasticity, responsible for the variability of the mating strategies observed in different environments. Given a major shift in the ecological niche between wolves and domestic dogs that occurred within a short period (in the evolutionary timescale), FRDs provide a unique model for studying the effect of ecological conditions on mating behavior.

Our result, showing polygynandrous mating system in FRDs in contrast with predominant social monogamy in wild canids, is consistent with the hypothesis that ecological conditions found in anthropogenic environments, that is, abundant/accessible food and high population density, can foster the evolutionary transition from monogamy to polygamy. Moreover, our results confirm that the predictions of the “polygyny threshold model,” initially developed to study evolution of birds (Orians 1969), can be verified in mammals as well, particularly in canids (Macdonald et al. 2019). In large canids like gray wolves, reproduction is energetically very costly (Creel and Creel 1991; Moehlman and Hofer 1997) and, in most wolf packs, it involves only a dominant breeding pair even in the presence of other sexually mature members (Mech and Boitani 2003). It is likely that dominant wolves often suppress the reproduction of mature subordinates in order to increase the amount of food available to their own puppies (Creel and Creel 1991; Derix et al. 1993).

The need for paternal care in canids depends on resource availability, as females may raise pups with less contribution from males if resources are abundant (Macdonald et al. 2019). If resources are scarce or difficult to obtain, paternal care is necessary for successful rearing of the litter and cannot be shared between multiple litters, thus favoring monogamy. In anthropogenic environments that are a typical habitat of FRD populations, resource availability may be sufficient to allow mothers to meet the high energetic costs of reproduction more easily, even when mating with polygynous males who provide reduced or no paternal care (Lord et al. 2013; Coppinger and Coppinger 2016). Moreover, access to human-derived food may have relaxed social constraints on the reproduction of subordinate pack members, resulting in packs comprising of a higher number of breeding members of both sexes (Cafazzo et al. 2014; Bonanni et al. 2017). The reduced need for paternal care makes polygamy advantageous to both males and females through increasing the genetic diversity of offspring (Neff and Pitcher 2005). Moreover, once paternal care is not necessary for offspring survival, males can maximize their reproductive success by increasing the number of mates. Although female reproduction rate is limited by resource availability rather than the number of mates, polygamy may allow females to increase their reproductive fitness as well. Specifically, polygamy can increase the average fitness of their offspring as a result of their higher genetic variability and enhanced fecundity, as well as via sperm competition and/or cryptic mechanisms of female choice that would allow females to obtain indirect fitness benefits for their offspring (Jennions and Petrie 2000; Simmons 2005; Gerlach et al. 2012).

However, in changeable anthropogenic environments, there may be times when food sources become scarce and helping by group members may be required for mothers to successfully raise puppies. This kind of environmental variability may explain why cooperation in raising puppies, including paternal care, has been observed in several packs of FRDs (Pal 2005; Paul and Bhadra 2018). Therefore, it may be hypothesized that the social organization and mating system of dogs, as those of wild canids, are shaped by abundance, distribution, and type of food resources (Macdonald and Carr 1995). In FRDs, pack members can also cooperate in defending puppies against predators (Pal 2005; Paul and Bhadra 2018), and so variation in the abundance of potential predators may also contribute to variation in the degree of cooperation observed.

One of the consequences of the polygynandrous mating system is the presence of a large number of maternal and paternal half-siblings, resulting in a broader network of relatives in the population. While the number of offspring a female can have (and thus the number of full-siblings or maternal half-siblings her offspring can have) depends on the resource availability, the number of offspring of a male (and thus the number of paternal half-siblings his offspring can have) can be much larger compared with the number of full-siblings in a monogamous mating system. Therefore, polygynandrous mating system results in a larger number of relatedness links in the population compared with a conspecific/congeneric population with a monogamous mating system.

In this study, we found that all but two of the genetically identified individuals were linked by a network of kinship relationships (Figure 3). We also observed the presence of relatives within packs, including half-siblings and mother–offspring pairs, which implied retention of adult offspring in natal groups. This is consistent with behavioral observations, showing that, in this population, packs were usually formed through retention of a considerable number of weaned pups (Bonanni and Cafazzo 2014; Bonanni et al. 2017). Retention of pups was also observed in other FRD populations (Gipson 1983; Daniels and Bekoff 1989; Paul and Bhadra 2018). In wolves, retention of pups in their natal packs is common, but some subadult individuals disperse from the natal pack at the onset of sexual maturity, with the number of dispersers being regulated by within-pack competition for food (Mech and Boitani 2003). Similarly, in the dog population studied, the presence of related individuals in different packs indicated short-distance dispersal, although the factors affecting it were not explored.

In wolves, group living increases individual fitness through benefits resulting from cooperative breeding, collective defense of dens within territories, and cooperative hunting of large prey (Stahler et al. 2013; MacNulty et al. 2014; Smith et al. 2015). In wolf family packs, participation by pups’ older siblings in cooperative breeding presumably increases their inclusive fitness, which may explain why wolf packs are usually comprised of close relatives. In FRDs, the fitness consequences of grouping have not been investigated yet, although it has been suggested that one of the most important benefits of grouping may be the possibility of defending resources collectively (Macdonald and Carr 1995, Bonanni and Cafazzo 2014). In the study population, individuals displayed scent-marking behavior typical of wolves (which was not restricted to the highest-ranking male and female, although its rate was positively affected by dominance rank; Cafazzo et al. 2012), and larger packs usually outcompeted smaller ones in interpack conflicts over food and space (Bonanni et al. 2011). Moreover, cooperation in raising puppies, which has been observed primarily among closely related females, may provide them with inclusive fitness benefits (Paul and Bhadra 2018). Our finding that dog packs include relatives suggests that inclusive fitness benefits may have contributed to the evolution of group living in FRDs and might support the suggestion that these animals are more cooperative than is usually supposed (Bonanni et al. 2017; Paul and Bhadra 2018).

In conclusion, we have provided the first genetic evidence that the mating system of FRDs is characterized by polygynandry in contrast with the predominantly monogamous mating system of gray wolves and other wild canids. This result suggests that the transition from monogamy to polygynandry in dogs could have been associated with the domestication process. Specifically, we suggest that access to anthropogenic food allowed female FRDs to bear the energetic costs of reproduction, thus reducing their reliance on paternal care, increasing tolerance among breeding females, and reducing benefits of interfering with other females’ reproduction. At the same time, high population density resulting from access to anthropogenic food provided both male and female FRDs with higher chances of polygamy. The relaxation of conditions thought to be associated with the stability of social monogamy in cooperative breeders (Creel and Creel 1991; Moehlman and Hofer 1997; Macdonald et al. 2019) was thus the likely driver of the transition toward a polygamous mating system in dogs.

## FUNDING

This work was supported by the University of Lincoln, the Polish National Agency for Academic Exchange—NAWA (Polish Returns Fellowship PPN/PPO/2018/1/00037 to M.P.), and Polish National Science Centre (grant 2019/34/E/NZ8/00246 to M.P.).

## Supplementary Material

arab011_suppl_Supplementary-Material

## Data Availability

Analyses reported in this article can be reproduced using the data provided by Natoli et al. (2021).
