# Supplementary material for: Genetic inference of the mating system of free-ranging domestic dogs
Source: Behav Ecol. 2021 Apr 2;32(4):646–56. doi: 10.1093/beheco/arab011 (PMC8444980; doi:10.1093/beheco/arab011)
Supplement: arab011_suppl_Supplementary-Material [file arab011_suppl_supplementary-material.pdf]

Supplementary Information

Genetic inference of the mating system of free-ranging domestic dogs

Eugenia Natoli, Roberto Bonanni, Simona Cafazzo, Daniel S. Mills, Dominique Pontier and Małgorzata Pilot

Supplementary Results

To understand the evolutionary relationships between the Italian free-ranging dog (FRD) population studied and other dog populations, we merged genome-wide SNP genotypes produced in this study with genome-wide SNP genotypes of pure-bred dogs and free-ranging dogs from publicly available datasets (Vaysse et al. 2011, Pilot et al. 2015). The merged genotypes were pruned using PLINK software (www.cog-genomics.org/plink/1.9; Chang et al. 2015) to remove SNPs with less than 10% of missing data and those in strong linkage disequilibrium (r^2>0.5). We carried out the Principal Component Analysis (PCA) using the Eigenstrat package of the Eigensoft software (Price et al. 2006).

The PCA showed that the Italian FRD population studied does not constitute a mixture of breeds and is genetically similar to other Eurasian FRD populations (see Figure 2 in the main text). Specifically, this population showed closest genetic proximity to other European populations included in the analysed dataset (from Bulgaria, Poland and Slovenia), but did not form one cluster with them. The PCA plot was consistent with that in Pilot et al. (2015; Supplementary Figure 8B) in showing the genetic distinctiveness of FRDs from pure-bred dogs. Pure-bred dogs formed three clusters distinct of FRDs, consisting of (1) East Asian and Arctic breeds, (2) breeds of European origin and (3) German shepherds. The reason for this last breed forming a distinct cluster is discussed in Pilot et al. (2015). As in that study (Pilot et al. 2015, Supplementary Figure 8C), we found that a few individual free-ranging dogs cluster with pure-bred dogs, suggesting gene flow between these groups, probably via stray pure-bred dogs joining FRD packs. However, we did not observe this in the Italian FRD population studied. Although owned dogs have been occasionally abandoned in the area inhabited by the FRD packs studied, these dogs were not necessarily pure-bred or mixed-bred, and could instead originate from the FRD stock. In conclusion, this analysis shows that the Italian population studied does not constitute a mixture of breeds and is representative of Eurasian FRD populations.

References

Chang, C.C., Chow, C.C., Tellier, L.C.A.M., Vattikuti, S., Purcell, S.M., Lee, J.J. 2015. Second-generation PLINK: rising to the challenge of larger and richer datasets. GigaScience, 4, s13742-015.

Pilot, M., Malewski, T., Moura, A. E., Grzybowski, T., Oleński, K., Ruść, A., et al. (2015). On the origin of mongrels: evolutionary history of free-breeding dogs in Eurasia. Proc. R. Soc. B, 282, 20152189.

Price, A. L., Patterson, N. J., Plenge, R. M., Weinblatt, M. E., Shadick, N. A., Reich, D. (2006). Principal components analysis corrects for stratification in genome-wide association studies. Nature Genet., 38, 904-909.

Vaysse, A. Ratnakumar, A., Derrien, T., Axelsson, E., Pielberg, G. R., Sigurdsson, S., et al. 2011. Identification of genomic regions associated with phenotypic variation between dog breeds using selection mapping. PLoS Genet., 7, e1002316.

Supplementary Table S1. Comparison of kinship relationships identified in COLONY, PRIMUS and CERVUS. "Group" represents full-sibling (FS) or half-sibling (HS) group identified in COLONY, P(inc.) represents inclusion probabilities for all individuals from a group listed in the first column. Relationships among individuals from each group inferred in PRIMUS and CERVUS are presented in subsequent columns. "Parentage" represents sampled mothers and fathers of the FS groups or single individuals identified in COLONY and the probabilities of these parentage assignment. The following columns indicate whether parents identified in COLONY were confirms in PRIMUS and CERVUS. "PO" denotes a parent-offspring relationship.

	group kinship				parentage (Colony)				parentage confirmation	
Group	Colony	P(Inc.)	Primus	Cervus	Mother	P-value	Father	P-value	Primus	Cervus
ID02,ID03,ID04,ID05,ID07,ID08,ID09	FS	1	FS	FS	ID1	1	ID37	1	yes	yes
ID12, ID13	FS	1	FS	FS	ID11	1	-		FS	yes
ID35, ID39	FS	1	FS	HS	ID48	1	-		yes	yes
ID15,ID16,ID17,ID18,ID22,ID23,ID42,ID44	FS	0.999	FS	PO groups						
ID01, ID34	FS	1	FS	-						
ID24,ID30,ID32,ID41,ID47,ID48	FS	1	FS and PO groups	PO groups						
ID36, ID40	FS	1	FS	-						
ID1, ID38, ID45	HS	0.068; 1	HS	-						
ID34, ID38, ID45	HS	1; 0.249	HS	-						
ID11, ID36	HS	1	HS	-						
ID11, ID40	HS	1	HS	-						
ID20, ID28, ID37	HS	1	HS	-						
ID19, ID26, ID27, ID31	HS	1	HS	-						
ID31					ID24	1	-		yes	yes
ID50					ID30	1	-		FS	yes
ID25					-		ID37	1	yes	yes
ID26					-		ID51	0.5	yes	yes

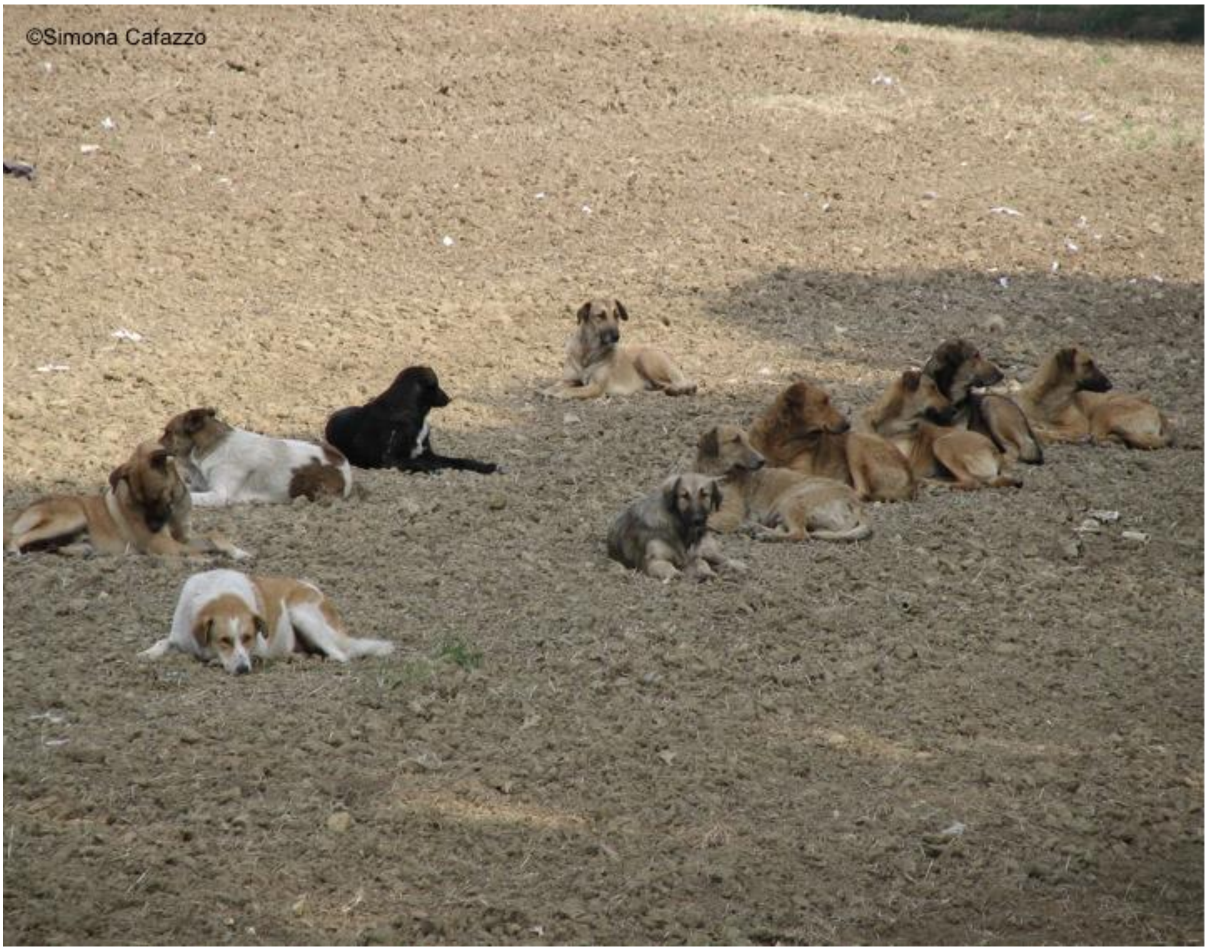

Supplementary Figure S1. Phenotypes of dogs from the study population.
